# Navigating the Storm: Myxedema Coma-Induced Cardiomyopathy Culminating in Refractory Cardiogenic Shock

**DOI:** 10.7759/cureus.61615

**Published:** 2024-06-03

**Authors:** Saimanoj Guntaka, Allan Lin, Suhwoo Bae, Michael Vaysblat, Matthew Pierce

**Affiliations:** 1 Internal Medicine, Northwell Health, Manhasset, USA; 2 Cardiology, Northwell Health, Manhasset, USA

**Keywords:** non-ischemic cardiomyopathy, cardiac intensive care unit, orthotopic heart transplant, va-ecmo, hypothyroid myxedema coma

## Abstract

Myxedema coma is a rare and life-threatening consequence of severe hypothyroidism, often precipitated by physiologic stressors. While cardiac manifestations are common, they are typically reversible with prompt treatment. Here, we report a case of a 23-year-old male with untreated hypothyroidism who presented with myxedema coma-induced cardiomyopathy leading to refractory cardiogenic shock requiring veno-arterial extracorporeal membrane oxygenation (VA-ECMO) and, ultimately, orthotopic heart transplantation (OHT). Our case highlights a rare occurrence of refractory shock necessitating mechanical support as a bridge to a cardiac transplant. We emphasize early recognition, aggressive management, and a low threshold to escalate care to mitigate the high mortality associated with myxedema coma.

## Introduction

Myxedema coma is a severe complication of untreated hypothyroidism, often precipitated by physiological stressors like infection or trauma. This life-threatening condition is challenging to identify and can result in significant organ dysfunction with a high mortality rate [1]. Patients may exhibit symptoms of hypothyroidism, including dry skin, weight gain, non-pitting edema, and cold intolerance [2]. Progression to lethargy and coma can occur along with involvement of the neurological, cardiovascular, respiratory, renal, and gastrointestinal systems due to the abundance of thyroid hormone receptors [3]. Cardiac manifestations occur frequently and encompass abnormal heart rate and rhythm, alterations in myocardial contractility, and changes in vascular tone [4]. Timely intervention can often reverse these findings, although in rare cases, complications like chronic heart failure or cardiogenic shock may ensue. We report a unique case of myxedema coma in a 23-year-old male who developed cardiogenic shock despite treatment, necessitating veno-arterial extracorporeal membrane oxygenation (VA-ECMO) and eventual orthotopic heart transplant (OHT).

## Case presentation

**Figure 1:**
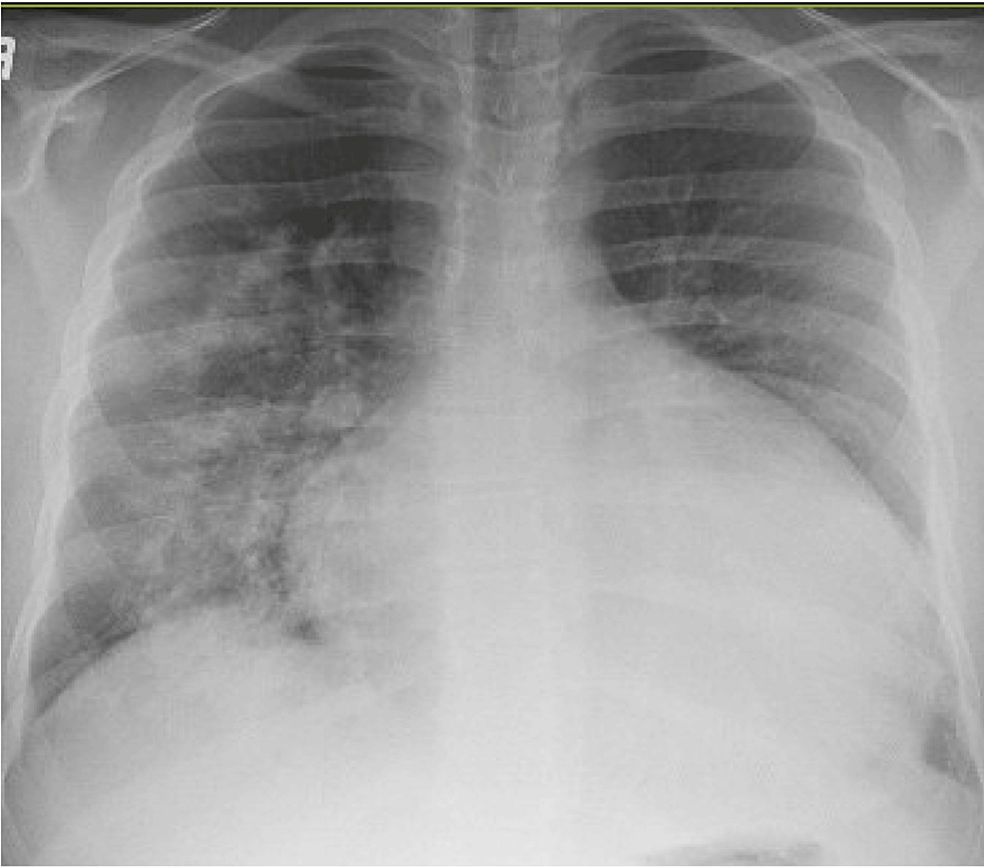
Chest X-Ray on admission Chest X-ray on admission showed prominent cardiomegaly and pulmonary congestion

A 23-year-old male with hypothyroidism presented to our hospital with worsening right upper quadrant abdominal pain for two weeks. Previous records indicated longstanding hypothyroidism, evidenced by a significantly elevated thyroid stimulating hormone (TSH) level of 100 uIU/mL (reference range: 0.27 - 4.2 uIU/mL). The patient was aware of his diagnosis; however, he was unable to start thyroid hormone supplementation and was lost to follow-up. On admission, he was hypotensive to 90/56 with a heart rate of 104 beats per minute (BPM) with a normal respiratory effort and oxygen saturation. Physical examination revealed signs of fluid overload, including pitting edema, elevated jugular venous distension, and bilateral lung crackles. Shortly thereafter, the patient experienced pulseless electrical activity (PEA) arrest, prompting initiation of advanced cardiac life support (ACLS) and return of spontaneous circulation (ROSC) after three minutes. Laboratory testing revealed elevated lactate, liver function tests, high-sensitivity cardiac troponin, and pro-brain natriuretic peptide (Pro-BNP; Table 1). A chest X-ray performed upon admission revealed cardiomegaly and pulmonary congestion (Figure 1).

**Table 1:** Laboratory results Initial results indicative of poor perfusion with myocardial injury and thyroid abnormalities

Variables	Serum level	Reference range
Lactate	7.2 mmol/L	0.5 - 2 mmol/L
Aspartate transferase (AST)	442 U/L	4 - 40 U/L
Alanine transaminase (ALT)	353 U/L	4 - 41 U/L
Cardiac troponin-T	273 ng/L	<50 ng/L
Pro-brain natriuretic peptide (pro-BNP)	6100 pg/mL	<299 pg/mL
Thyroid-stimulating hormone (TSH)	857 uIU/mL	0.27 - 4.2 uIU/mL
Triiodothyronine (T3)	<20 ug/dL	80 - 200 ug/dL
Thyroxine (T4)	0.5 ug/dL	4.6 - 12 ug/dL
Cortisol	12.7 ug/dL	6 - 18.4 ug/dL

**Figure 2:**
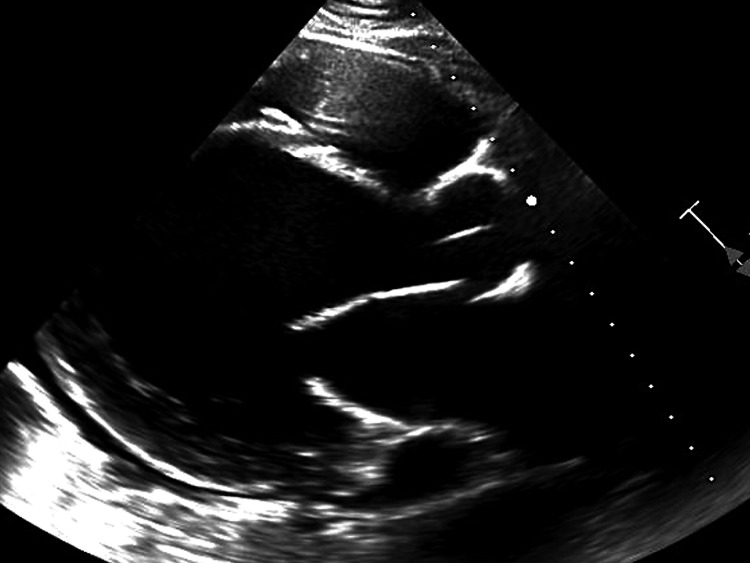
Transthoracic echocardiogram Parasternal long view demonstrating severe dilatation and global hypokinesis of the left ventricular cavity with an ejection fraction of 10% and mild pericardial effusion

He was admitted to the cardiac intensive care unit (CCU) and was found to be in a low-flow state, as evidenced by findings from the pulmonary artery catheter, which revealed a low cardiac index and elevated filling pressures. Additional bloodwork showed a normal cortisol level and elevated TSH level with markedly diminished total triiodothyronine (T3) and thyroxine (T4) levels (Table 1). A transthoracic echocardiogram (TTE) revealed severe biventricular dysfunction characterized by global hypokinesis, left ventricular ejection fraction of 10%, and a mild pericardial effusion without evidence of tamponade (Figure 2). Despite initial interventions, including initiation of vasopressors, inotropes, and placement of an intra-aortic balloon pump (IABP), the patient's shock indexes continued to worsen. Consequently, a cardiogenic shock call was initiated as per our institutional protocol, leading to the decision to implement VA-ECMO with atrial septostomy.

**Figure 3:**
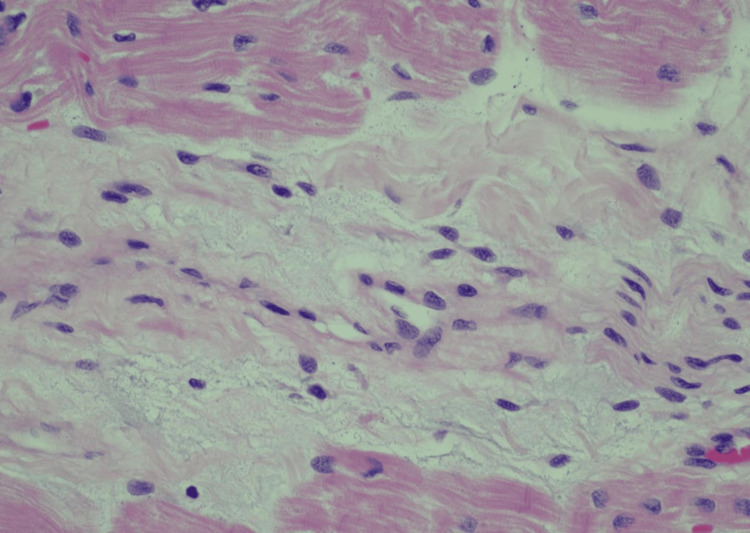
Cardiac histopathology Histopathology from the explanted heart demonstrating extensive fibrosis

**Figure 4:**
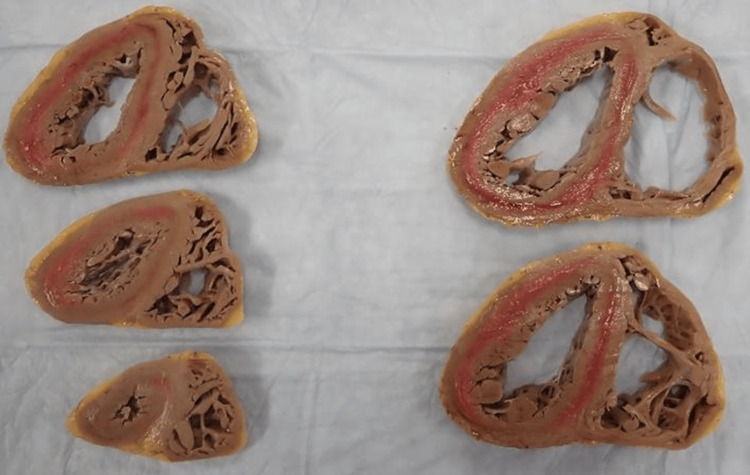
Explanted cardiac tissue Cross-sections from the explanted heart showing left ventricular hypertrophy

Due to the severe hypothyroidism, there was a high suspicion of myxedema coma-induced cardiomyopathy, and endocrinology was consulted. High-dose levothyroxine (400mcg intravenously followed by 100mcg daily), 10mcg T3 intravenously, and 100mg hydrocortisone every eight hours were initiated. Despite progressive improvement in thyroid function studies over seven days of replacement therapy, the patient remained in refractory cardiogenic shock and was listed for OHT on hospital day eight. Due to bleeding complications from his cannulation sites and new-onset melena, his OHT was expedited. The cardiac histopathology of his explanted heart reveals extensive fibrosis (Figure 3) and significant left ventricular hypertrophy (Figure 4). He was discharged on levothyroxine 137mcg daily and liothyronine 5mcg twice daily. His post-operative course was uneventful with close follow-ups with cardiology and endocrinology. Further workup of his hypothyroidism did not show any signs of radiation or medication-induced thyroid disease. Furthermore, he tested negative for antibodies to thyroid peroxidase (TPO) and thyroglobulin (TG), and a thyroid ultrasound indicated a normal-sized thyroid gland. He was ultimately transitioned to monotherapy with levothyroxine 150mcg daily with an improvement of his thyroid function tests.

## Discussion

Myxedema coma primarily affects elderly females and is commonly triggered by severe illness, trauma, or cessation of thyroid supplements. Diagnosis can be challenging due to diverse clinical manifestations such as hypothermia, hypercapnia, hypoxemia, and shock. If left untreated, myxedema coma can result in multi-organ failure, infection, and death. Due to the profound impact of thyroid disorders on the cardiovascular system, myxedema coma may also manifest with signs and symptoms of heart failure.

While the precise mechanisms are not fully understood, various theories propose that thyroid hormones exert direct effects on peripheral vasculature and cardiac myocytes. Hyperthyroidism can enhance volume return to the heart by concomitantly reducing arterial tone and augmenting venous return, thereby enhancing cardiac preload. The renin-angiotensin-aldosterone axis is also activated, which plays a role in modulating sodium and calcium homeostasis to further supplement intravascular volume [4,5]. Conversely, inadequate thyroid hormone levels have significant consequences, including diminished cardiac output and conduction abnormalities [5-8]. Without sufficient regulation of sodium and calcium levels, blood return to the heart may be compromised, resulting in decreased stroke volume.

Thyroid hormones exert their effects at the cellular level on cardiac myocytes. Triiodothyronine enhances the expression and synthesis of proteins involved in myocyte contraction [5]. Furthermore, it positively influences cytosolic calcium levels and ion channels, resulting in enhanced systolic contraction and improved diastolic relaxation [5]. In the absence of these hormones, there is decreased activity of contractile mechanisms, ultimately leading to reduced cardiac output. Additionally, thyroid hormones play a role in modulating the sympathetic nervous system, contributing to increased heart rate and stroke volume upon activation. A lack of these hormones leads to an imbalance between alpha and beta-adrenergic outputs, exacerbating the decline of cardiac output.

Myxedema coma is primarily diagnosed clinically, with thyroid function tests serving as a supplementary diagnostic tool. In our case, our patient had the clinical signs of myxedema coma, and bloodwork revealed diminished thyroid hormones. Concurrently, he presented in cardiogenic shock, necessitating vasopressors and inotropic support. Literature review yielded several reports of thyroid-induced heart failure; however, none documented patients in refractory cardiogenic shock despite appropriate thyroid hormone replacement [9-11].

Given the considerable mortality associated with myxedema coma, timely intervention and vigilant monitoring in an intensive care unit setting are imperative. While thyroid hormone replacement constitutes the cornerstone of treatment, addressing concurrent metabolic disturbances is equally important [2]. Due to the frequent coexistence of hypoadrenalism, adjunctive glucocorticoid therapy is recommended [12]. Despite receiving thyroid hormone replacement and glucocorticoids, our patient's persistent refractory shock necessitated the initiation of VA-ECMO and, ultimately, OHT.

## Conclusions

Progression of severe hypothyroidism to myxedema coma can lead to life-threatening complications stemming from severe organ dysfunction. Cardiac involvement can lead to complications such as ventricular arrhythmias, heart failure, cardiogenic shock, and death. Diagnosis is challenging due to the diverse symptomatology, underscoring the importance of considering thyroid abnormalities in patients presenting with cardiac abnormalities. Here, we present a unique and complex case involving refractory myxedema coma-induced cardiogenic shock in a young male with hypothyroidism. Despite aggressive thyroid hormone replacement, his shock state progressed, requiring VA-ECMO as a bridge to OHT. We emphasize timely identification and intervention to potentially mitigate and reverse complications from myxedema coma. However, we highlight that despite treatment, clinical deterioration can progress, and physicians should hold a low threshold for escalation of care.
